# Analysis of Plasma EBV-DNA and Soluble Checkpoint Proteins in Nasopharyngeal Carcinoma Patients after Definitive Intensity-Modulated Radiotherapy

**DOI:** 10.1155/2019/3939720

**Published:** 2019-05-05

**Authors:** Yanyun Ruan, Wei Hu, Wanhong Li, Hongsheng Lu, Huamin Gu, Ying Zhang, Chumeng Zhu, Qi Chen

**Affiliations:** ^1^Precision Medicine Center, Taizhou Central Hospital (Taizhou University Hospital), Taizhou, Zhejiang, China; ^2^Department of Radiotherapy, Taizhou Central Hospital (Taizhou University Hospital), Taizhou, Zhejiang, China; ^3^Department of Laboratory, Taizhou Central Hospital (Taizhou University Hospital), Taizhou, Zhejiang, China; ^4^Department of Pathology, Taizhou Central Hospital (Taizhou University Hospital), Taizhou, Zhejiang, China

## Abstract

**Background:**

Tumor immunotherapy and immunological checkpoint-related proteins are research hotspots. Intensity-modulated radiotherapy (IMRT) is the main treatment for nasopharyngeal carcinoma (NPC). Hence, the evaluation of its effect is very important. The aim of this study was to assess the relationship between the concentrations of soluble checkpoint proteins, plasma EBV-DNA, and cytokines in NPC patients treated with IMRT.

**Methods:**

In this study, the plasma samples of 37 NPC patients and 40 healthy controls were collected. Luminex MAGPIX was used to detect the concentrations of 32 plasma targets, including soluble programmed cell death 1 (sPD-1). RT-qPCR was used to measure EBV-DNA.

**Results:**

The concentrations of 33 plasma targets were detected in NPC patients before and after IMRT to explore the changes after IMRT. The results showed that IMRT could increase the expression of sPD-1 and significantly reduce the level of EBV-DNA in the plasma of NPC patients. The expression level of sPD-1 in TNM I/II patients was significantly higher than that in III/IV patients. Besides, the concentrations of 12 other targets were significantly different after IMRT, including LAG-3, PD-L1, TIM-3, IFN-*γ*, IL-12p70, IL-1*β*, IL-5, IL-6, TNF-*α*, IL-10, IL-17A, and IL-22. High sPD-1 patients had longer survival than those with low sPD-1. Also, patients with lower EBV-DNA and TNM grades I and II/III had longer survival than those with higher EBV-DNA or TNM IV.

**Conclusions:**

This study demonstrated that the concentration of sPD-1 was significantly increased and EBV-DNA was significantly reduced in the NPC patients after IMRT. Plasma EBV-DNA level was a highly specific and sensitive biomarker for NPC diagnosis. Both sPD-1 expression and EBV-DNA concentration in plasma were related to the survival of patients.

## 1. Introduction

Nasopharyngeal carcinoma (NPC) is a malignant tumor of the upper and lateral wall of the nasopharyngeal cavity, which exhibits marked racial and geographical differences. It is prevalent in northern Africa, southeast Asia, and southern China. The age-standardized rates (ASRs) of NPC are as high as 20/100,000 person-year in southern China, but less than 1/100,000 in most areas worldwide [1].

As nodal metastasis is frequently found in probably 75% of NPC patients, blood biomarkers for early diagnosis of NPC are urgently needed [2]. The critical relationship between cytokines and immunity is involved in the growth, survival, inflammation, and development of tumor cells.

Several cytokines and proteins have been identified as potential markers for diagnosis and prognosis of NPC. Serum level of interleukin-6 (IL-6) was reported to predict overall survival (OS), disease-free survival (DFS), distant metastasis-free survival (DMFS), and lung metastasis-free survival (lung-MFS) in NPC patients [3]. High plasma soluble CD40 ligand (sCD40L) level was significantly related to stage progression and poor survival of NPC patients [4]. After extensive literature review, we identified 29 cytokines, CD molecules, and soluble proteins that are closely related to tumors, including Th1/Th2 and Treg-related factors. The Epstein-Barr virus (EBV) is linked to host immune cells and NPC risk. Plasma EBV-DNA was identified as a useful marker for the detection, monitoring, and prognosis of NPC [5, 6].

The immune checkpoint regulates the immune response of T cells through a dual signaling mechanism and plays a vital role in maintaining autoimmune tolerance. Immunological checkpoints have achieved good clinical results as drug targets, especially programmed death 1 (PD-1) and PD-L1. PD-1, due to its diverse roles in regulating immune responses during malignancy, has become an important focus in tumor immunotherapy in recent years. PD-1 pathway causes immune suppression of tumors through downregulation of effector T cells [7].

sPD-1 is a soluble form of PD-1, which competitively blocks the regulatory properties of PD1/PD-L1, and thus improves antitumor immunity. In preclinical studies, sPD-1 was detectable in healthy individuals, and its levels were increased in patients with autoimmune diseases and cancer [8]. Given the convenience of plasma extraction, widespread suppression of all combinations of PD-1/PD-L1, and stability of plasma sPD-1 level, sPD-1 was considered as a new biomarker and effective potential immunotherapy target.

In the present study, expression levels of 32 plasma checkpoint proteins, cytokines, and EBV-DNA were detected in NPC patients before and after intensity-modulated radiotherapy (IMRT), which is the preferred treatment due to the radiosensitivity of NPC.

## 2. Materials and Methods

### 2.1. Study Population

A total of 37 patients with NPC who received IMRT in Taizhou Central Hospital (Zhejiang, China) and 40 healthy volunteers were enrolled in this study between July 14, 2015, and July 28, 2016. Patient data including age, gender, date of initial diagnosis, pathological diagnosis, TNM stage, treatment regimen, and date of death or last follow-up was collected (Table 1). There was no significant difference in age and gender between the NPC patients and healthy controls. The disease stage was determined according to the seventh TNM staging system of the International Union for Cancer Control (UICC) and the American Joint Committee for Cancer (AJCC).

Of the 37 cases, three (8.11%) were stage I, 13 (35.14%) were stage II, 17 (45.95%) were stage III, and four (10.81%) were stage IV. All the patients were followed-up, and the average of follow-up period was 29.0 months (range, 2.4-42.7 months). During the entire follow-up period, there were five cancer-related deaths (13.6%) including one, two, and two patients with stages II, III, and IV, respectively.

### 2.2. Samples and Methods

Plasma samples of patients and controls were prepared from the peripheral blood by centrifugation at 3000 rpm for 10 minutes and then stored at -80°C until use. A total of 32 targets including soluble checkpoint proteins and plasma cytokines were detected by Immuno-Oncology Checkpoint 14-Plex Human ProcartaPlex™ Panel 1 (eBioscience, Carlsbad, USA) and ProcartaPlex Human Th1/Th2/Th9/Th17/Th22/Treg cytokine kit (eBioscience, Carlsbad, USA) using Luminex MAGPIX. EBV-DNA was extracted from plasma and measured by RT-qPCR using ABI 7500 and EBV nucleic acid qPCR detection kit (Daan gene, Guangzhou, China). All the experiments were conducted as per the manufacturer's instructions.

### 2.3. Statistical Analysis

Statistical analysis was performed with SPSS17.0 software (IBM, Armonk, NY, USA), and GraphPad Prism 6 (GraphPad® Software, Inc., San Diego, CA, USA) was used to generate the graphs. Differences in the 32 targets and EBV-DNA between NPC patients before and after IMRT were analyzed with paired-samples t-test, and Wilcoxon Signed Rank test was used when the data was skewed. Independent-samples t-test and Mann-Whitney U-test were used to compare the targets between NPC patients and controls. Survival probabilities were calculated using the Kaplan-Meier method, and the differences between survival curves were analyzed by the log-rank test.* P*<0.05 was considered significant.

## 3. Results

### 3.1. Soluble Checkpoint Proteins and Plasma Cytokine Levels in NPC Patients and Healthy Controls

**Figure 1:**
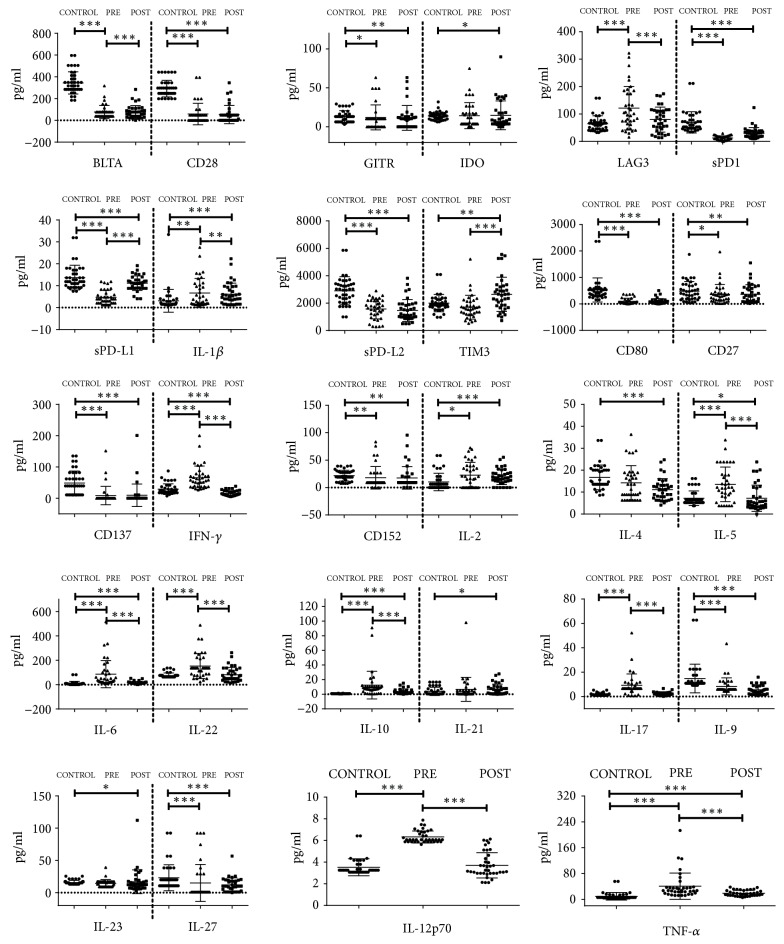
*Comparison of 32 plasma checkpoint protein expression in NPC patients before and after IMRT with controls* (*∗p* <0.05, *∗∗p*<0.01, and *∗∗∗p* <0.001).

Luminex was used to determine the expression levels of soluble checkpoint proteins and cytokines. Plasma proteins and cytokines were compared between NPC patients before IMRT and healthy controls, and the results showed that 24 out of 32 plasma proteins and cytokines had statistical significance (Table 2), namely, BTLA (*p≤0.001*), GITR (*p*=0.022), HVEM (*p≤0.001*), LAG-3 (*p≤0.001*), sPD-1 (*p≤0.001*), sPD-L1 (*p≤0.001*), sPD-L2 (*p≤0.001*), CD28 (*p≤0.001*), CD80 (*p≤0.001*), CD137 (*p≤0.001*), CD27 (*p*=0.049), CD152 (*p*=0.002), IFN-*γ* (*p≤0.001*), IL-12p70 (*p≤0.001*), IL-1*β* (*p*=0.002), IL-2 (*p*=0.027), IL-5 (*p≤0.001*), IL-6 (*p≤0.001*), TNF-*α* (*p≤0.001*), IL-10 (*p≤0.001*), IL-17A (*p≤0.001*), IL-22 (*p≤0.001*), IL-27 (*p≤0.001*), and IL-9 (*p≤0.001*) (Figure 1).

After IMRT, 24 out of 32 plasma proteins and cytokines were significantly altered in NPC patients as compared to healthy donors (Table 2). Of these, nearly half were significantly lower than controls after IMRT, including BTLA (*p≤0.001*), GITR (*p*=0.009), HVEM (*p≤0.001*), sPD-1 (*p≤0.001*), sPD-L1 (*p*=0.017), sPD-L2 (*p≤0.001*), CD80 (*p≤0.001*), CD137 (*p≤0.001*), CD27 (*p*=0.004), CD152 (*p*=0.003), IFN-*γ* (*p≤0.001*), and IL-4 (*p≤0.001*). The remaining factors were increased after IMRT as compared to controls, namely, IDO (*p*=0.019), TIM-3 (*p*=0.005), CD28 (*p≤0.001*), IL-1*β* (*p≤0.001*), IL-2 (*p≤0.001*), IL-5 (*p*=0.036), IL-6 (*p≤0.001*), TNF-*α* (*p≤0.001*), IL-10 (*p≤0.001*), IL-21 (*p*=0.029), and IL-23 (*p*=0.024) (Figure 1).

Plasma soluble checkpoint proteins and cytokines were also compared between pre- and post-IMRT groups, and 13 out of 32 showed statistical significance (Table 2). Nine of them were significantly reduced after IMRT, including IFN-*γ* (*p≤0.001*), IL-12p70 (*p≤0.001*), IL-1*β* (*p*=0.005), IL-5 (*p≤0.001*), IL-6 (*p≤0.001*), TNF-*α* (*p≤0.001*), IL-10 (*p≤0.001*), IL-17A (*p≤0.001*), and IL-22 (*p≤0.001*), while four proteins were increased, namely, LAG-3 (*p≤0.001*), sPD-1 (*p≤0.001*), sPD-L1 (*p≤0.001*), and TIM-3 (*p≤0.001*) (Figure 1).

### 3.2. Differences in EBV-DNA between NPC Patients and Healthy Controls

**Figure 2:**
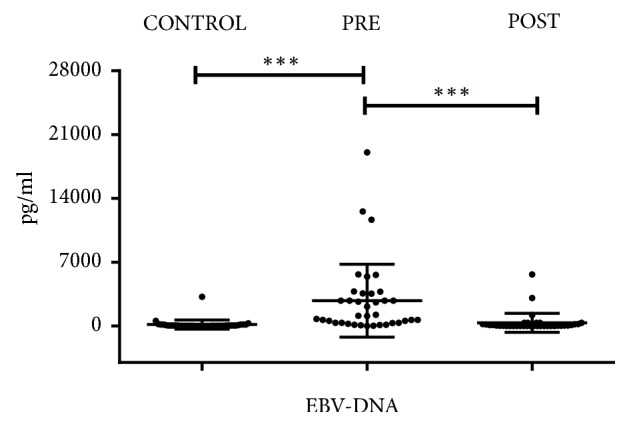
*Comparison of EBV-DNA level in NPC patients before and after IMRT with controls* (*∗p* <0.05, *∗∗p*<0.01, and *∗∗∗p* <0.001).

**Figure 3:**
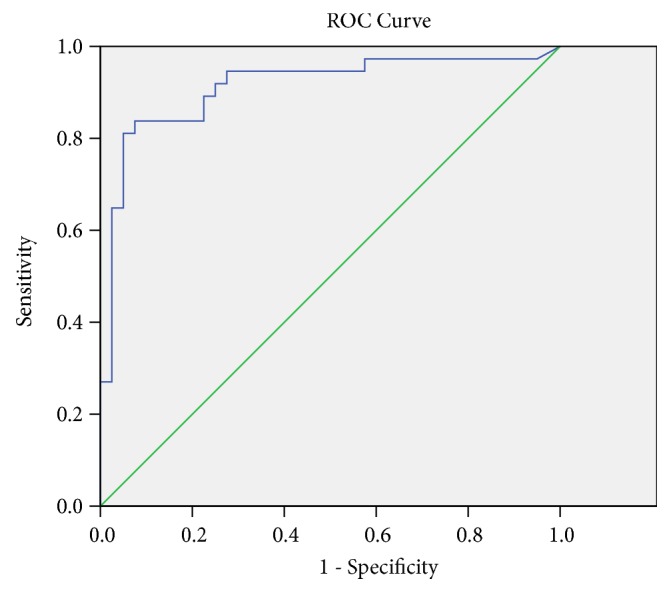
*ROC curve analysis of EBV-DNA levels for distinguishing between NPC patients and controls*. The area under the ROC curve is 0.912 (*p≤0.001*).

EBV-DNA was significantly higher in NPC patients before IMRT than after (2780±3998.8 vs. 366.7±1043.6 pg/ml,* p≤0.001*) and healthy controls (2780±3998.8 vs. 162.9±505.3 pg/ml,* p≤0.001*), but no difference was found between NPC patients after IMRT and healthy donors (Figure 2). This result indicated that IMRT was effective in decreasing EBV-DNA in NPC patients. ROC curve was used to distinguish the plasma levels of EBV-DNA between NPC patients and controls (Figure 3). The area under ROC curve was 0.912 (95% CI: 0.841-0.983,* p≤0.001*). Furthermore, there was no significant difference in EBV-DNA between TNM I/II and III/IV NPC patients.

### 3.3. Relationship between Plasma Proteins, Cytokines Expression Levels, and EBV-DNA in NPC Patients and Clinicopathological Stages

We compared the differences of plasma soluble proteins, cytokines and EBV-DNA between TNM I/II and III/IV NPC patients before IMRT, and only PD-1 showed significant difference. The expression level of PD-1 in TNM I/II patients was significantly higher than that in III/IV patients (16.0±5.9 vs. 9.7±8.1 pg/ml, p=0.012), suggesting that sPD-1 may be a biomarker for the development of NPC.

### 3.4. Correlation between Proteins, Cytokines and EBV-DNA in Plasma, and Prognosis of NPC Patients

**Figure 4:**
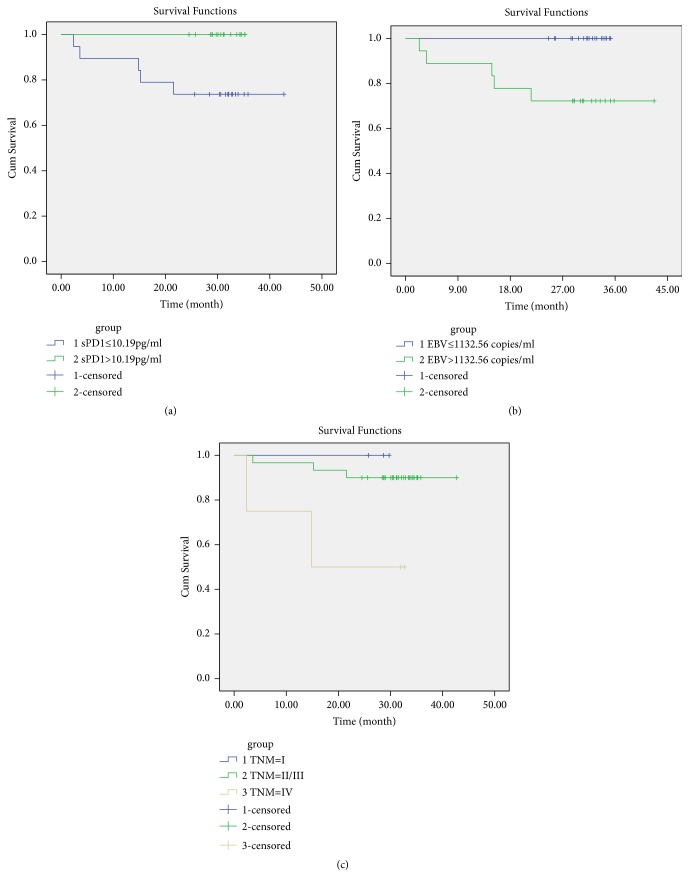
*Kaplan-Meier survival analysis of NPC patients*. (a) Comparison of the OS between patients with sPD-1 concentrations above and below the median (10.19 pg/ml,* p*=0.021). (b) Comparison of the OS between the patients with EBV-DNA levels above and below the median (1132.56 copies/ml,* p*=0.015). (c) Comparison of the OS between TNM I, II+III, and IV patients (*p*=0.026). Early stage, I; middle stage, II+III; late stage, IV.

To investigate the relationship between plasma targets and the clinical outcome of NPC patients, patient survival based on plasma proteins and cytokines, EBV-DNA, age, gender, and TNM stage was analyzed. Briefly, patient gender was stratified to male and female and age, and the detected targets were stratified to below and above the median values, and TNM stage was stratified to I, II+III, and IV, respectively. Patients with high sPD-1 (>10.19 pg/ml, n=18) had better survival than those with low sPD-1 (n=19,* p*=0.021) (Figure 4(a)). The survival time of the high sPD-1 patients was 31.0±3.2 months, and that of the low sPD-1 patients was 27.1±10.8 months.

Among other factors, only EBV-DNA and TNM stage were significantly associated with patient survival. Patients with higher EBV-DNA (>1132.56 copies/ml, n=18) had poor survival than those with lower EBV-DNA (n=19, 26.7±11.0 months vs. 31.1±3.3 months,* p*=0.015) (Figure 4(b)). Besides, patients with TNM I (n=3) and II/III (n=30) had significantly longer survival time than those with TNM IV (n=4, 28.1±2.1 months vs. 30.2±7.0 months vs. 20.5±14.6 months,* p*=0.026) (Figure 4(c)).

## 4. Discussion

NPC is a malignant tumor prevalent in southern China, especially in Guangdong, Guangxi, Hainan, Jiangxi, and Fujian provinces [9]. Though IMRT improves disease control and survival of NPC patients, early diagnostic and effective prognostic markers post-IMRT are urgently needed.

EBV-encoded RNAs could be detected in all NPC cells but not in normal nasopharyngeal epithelium which suggested that EBV activation is necessary for the pathogenesis of NPC. Latent and lytic cycles are the main forms of EBV infection, whereby EBV proteins evade host immune response using multiple strategies. Lytic EBV proteins block the secretion of many antiviral cytokines, such as BZLF1. BZLF1 could inhibit JAK/STAT signaling and IFN-*α* production by inducing SOCS3, which inhibits type I IFN response. Expression of LMP1, a latent EBV gene product, may recruit Treg to upregulate chemokine CCL20 via activation of NF-*κβ* signaling. CCL20 assists tumor cells in immune evasion by increasing the migration of CD4^+^Foxp3^+^ Tregs towards tumor location [6, 10–12].

Kanakary et al. [13] found that EBV in plasma had higher specificity and sensitivity than EBV in PBMCs in EBV^+^ disease, even among immunocompromised patients. In this study, we detected plasma EBV-DNA in pre and post-IMRT groups of NPC patients as compared to controls. Data showed that EBV-DNA was higher in NPC patients before IMRT than in groups after IMRT and controls. The results strongly suggested that EBV-DNA level in plasma was a highly specific and sensitive biomarker to differentially diagnose NPC patients from healthy individuals. Moreover, NPC patients with higher EBV-DNA in plasma before IMRT had a poorer prognosis than those with low EBV-DNA. This result was consistent with a previous study [2]. However, there was no disparity in EBV-DNA concentration between different TNM stages (TNM I/II vs. III/IV) for pre-IMRT NPC patients in the present study. In contrast, Prayongrat et al. noted a significant relationship between pre-IMRT EBV-DNA level and disease stage [14]. These discrepancies can be resolved by wider population study. Furthermore, the present study found that the concentration of EBV-DNA in NPC patients after IMRT was significantly correlated with locoregional failure, distant metastasis, and death. Post-IMRT EBV-DNA was suggested to be a predictor for various phases of IMRT [5, 14].

PD-1 is a member of the CD28/B7 family and acts as an immune checkpoint in various malignant tumors. PD-1 gene is characterized by the presence of alternative mRNA splicing mechanisms that encode both membrane-bound (flPD-1, PD-1 Deltaex2, PD-1 Deltaex2, 3, and PD-1 Deltaex2, 3, 4) and soluble (PD-1 Deltaex3) isoforms [15]. The activation of PD1/PD-L1 pathway leads to T cell tolerance, exhaustion and apoptosis, and enhancement of immunosuppressive Treg cell function, by which tumors evade immune surveillance [7]. Since tumor-cell surface PD-L1 is upregulated by local high-dose radiotherapy, concomitant PD-1 inhibition was recommended in radio-immunotherapy [16, 17]. This therapy was found to be effective in murine models and some tumor case reports [17–20].

sPD-1, which lacks exon 3, is generated by mRNA alternative splicing. Preclinical studies hypothesized that sPD-1 blocks the regulatory properties of PD-1/PD-L1, followed by restoration of T cell function, and proliferation and enhancement of immune-mediated tumor control [8]. Mice transfected with sPD-1 showed a strong antitumor immune response with delayed tumorigenesis and suppressed and regressed tumor progression [21, 22].

In this study, sPD-1 was demonstrated to have a significant relationship with TNM stage and prognosis of NPC patients after IMRT. sPD-1 concentration was significantly increased in patients after IMRT. Patients with higher sPD-1 had a longer survival time than those with low sPD-1. This result suggested that sPD-1 could improve antitumor immunity. Few clinical studies have analyzed the relationship between sPD-1 and clinicopathological characteristics of patients with malignancy. In Chang's [23] research, a high level of sPD-1 was correlated with a favorable OS and DFS, indicating that sPD-1 was an independent prognostic factor for patients with hepatocellular carcinoma. An increase in sPD-1 during erlotinib treatment was associated with prolonged PFS and OS in non-small-cell lung cancer [8]. In contrast, sPD-1 levels did not indicate adverse outcome in patients with advanced pancreatic cancer [24]. The relationship between sPD-1 and prognosis of various cancers needs further research.

The study of tumor microenvironment has been a research hotspot in recent years. LAG-3 suppresses T cell activation and cytokine secretion to maintain immune homeostasis, which shows a synergy between PD-1 and immune responses [25]. IFN-*γ* plays important antiviral, antitumor and immune regulation roles. The PD-1/PD-L1 pathway is one of the most representative IFN-induced immune escape systems. In Imai's research [26], pancreatic ductal adenocarcinoma cells promoted PD-L1 expression and epithelial-mesenchymal transition in the presence of IFN-*γ*. Elevated IL-6 levels were positively correlated with poor OS, DFS, DMFS, and lung-MFS in NPC patients [3]. IL-6 was found to be necessary for myeloid PD-L1 induction through a signal transducer and activator of STAT3-dependent mechanism in glioblastoma [27]. In orthotopic murine glioma models, the inhibition of IL-6 signaling led to reduced myeloid PD-L1 expression, diminished tumor growth, and increased survival. LAG-3, IFN-*γ*, and IL-6 levels were significantly reduced after IMRT for NPC in the present study. IL-10 suppressed the secretion of cytokines from Th1 cells and stimulated the proliferation and activity of CD8^+^ T cells in human immune microenvironment. IL-10 reduced macrophage activation and subsequent Th17 cell responses by inhibiting several cytokines (including IL-6) and the common IL-12p40 pathway shared by IL12 and IL-23 [28]. Therefore, numerous cytokines and soluble proteins constitute a complex and variable tumor microenvironment. In this study, we detected several immunological factors in pre- and post-IMRT groups of NPC patients. Nine soluble checkpoint proteins were dramatically reduced, including TIM-3, IFN-*γ*, IL-12p70, IL-1*β*, IL-5, TNF-*α*, IL-10, IL-17, and IL-22, while two (sPD-L1; TIM-3) were observably increased after IMRT.

## 5. Conclusions

This study found that four soluble checkpoint proteins were increased while 10 were reduced in pre- and post-IMRT groups of NPC patients. Dramatically reduced EBV-DNA after IMRT was closely related with favorable OS. ROC curve suggested that EBV-DNA level in plasma was an effective diagnostic marker for NPC patients. Increased sPD-1 was correlated with early TNM stage and longer survival.

## Figures and Tables

**Table 1:** Characteristics of nasopharyngeal carcinoma patients.

	Patient (n=37)	Control (n=40)
Age (year)		
Median	53	50
Range	37-90	20-73
Gender		
Male	25	24
Female	12	16
Tumor status		
T_1_	10	
T_2_	13	
T_3_	12	
T_4_	2	
Nodal status		
N_0_	5	
N_1_	15	
N_2_	17	
Metastasis status		
M_0_	34	
M_1_	3	
TNM stage		
I	3	
II	13	
III	17	
IV	4	

**Table 2:** Changes in soluble checkpoint proteins and plasma cytokines before and after IMRT in NPC.

	Pre-IMRT radiotherapy	Post-IMRT radiotherapy	Control	*p1*	*p2*	*p3*
BTLA	77.0±59.6	76.5±58.9	344.1±102.3	0.948	≤0.001	≤0.001
GITR	12.1±16.0	11.5±15.9	13.3±7.4	0.709	0.022	0.009
HVEM	4.1±11.6	7.7±21.3	94.0±18.3	0.325	≤0.001	≤0.001
IDO	14.3±16.7	14.7±18.5	13.9±5.0	0.737	0.084	0.019
LAG-3	121.4±79.5	79.7±45.5	64.8±28.6	≤0.00	≤0.001	0.087
sPD-1	12.4±7.8	30.0±20.6	69.7±38.8	≤0.001	≤0.001	≤0.001
sPD-L1	4.7±3.2	11.1±3.3	13.7±5.6	≤0.001	≤0.001	0.017
sPD-L2	1571.6±719.3	1463.3±797.8	2901.9±1026.1	0.388	≤0.001	≤0.001
TIM-3	1702.8±867.8	2643.3±1243.8	2005.6±633.4	≤0.001	0.087	0.005
CD28	58.6±98.6	53.2±83.3	292.0±76.2	0.315	≤0.001	≤0.001
CD80	99.9±114.4	92.6±110.6	526.4±454.5	0.191	≤0.001	≤0.001
CD137	8.8±29.4	9.6±35.9	48.8±38.1	0.715	≤0.001	≤0.001
CD27	358.7±377.0	369.9±355.4	480.3±368.7	0.731	0.049	0.004
CD152	18.0±20.5	17.6±20.6	21.7±9.8	0.386	0.002	0.003
IFN-*γ*	65.6±37.4	15.2±8.8	29.2±16.9	≤0.001	≤0.001	≤0.001
IL-12p70	6.3±0.6	3.7±1.2	3.5±0.8	≤0.001	≤0.001	0.607
IL-13	14.0±12.3	17.7±9.8	14.7±8.9	0.154	0.566	0.070
IL-1*β*	6.6±6.7	6.1±5.1	3.1±5.2	0.005	0.002	≤0.001
IL-2	22.8±23.2	19.6±14.0	10.2±15.9	0.563	0.027	≤0.001
IL-4	14.2±7.8	11.2±5.0	16.7±5.7	0.782	0.119	≤0.001
IL-5	13.5±7.9	7.2±6.0	7.2±3.3	≤0.001	≤0.001	0.036
IL-6	86.2±111.7	15.3±11.2	9.0±17.6	≤0.001	≤0.001	≤0.001
TNF-*α*	40.8±40.7	19.0±8.9	9.6±11.8	≤0.001	≤0.001	≤0.001
GM-CSF	12.4±15.1	13.5±10.9	11.1±12.0	0.417	0.319	0.240
IL-18	57.6±79.1	49.8±28.0	63.4±42.5	0.405	0.076	0.104
IL-10	12.6±19.0	3.5±3.6	0.8±0.2	≤0.001	≤0.001	≤0.001
IL-17A	9.3±9.3	1.9±1.5	1.6±1.1	≤0.001	≤0.001	0.597
IL-21	6.7±16.4	6.3±7.5	4.1±5.7	0.208	0.182	0.029
IL-22	153.0±102.6	81.6±59.3	75.0±20.9	≤0.001	≤0.001	0.171
IL-23	14.3±6.0	17.0±18.4	15.6±3.4	0.904	0.127	0.024
IL-27	15.1±28.6	12.0±11.0	23.1±20.1	0.517	≤0.001	≤0.001
IL-9	8.3±7.0	5.5±3.9	14.8±11.8	0.209	≤0.001	≤0.001

*p1,* pre-IMRT and post-IMRT.

*p2,* pre-IMRT and control.

*p3, *post-IMRT and control.

## Data Availability

The data used to support the findings of this study are included within the article.

## References

[1] Tang L.-L., Chen W.-Q., Xue W.-Q. (2016). Global trends in incidence and mortality of nasopharyngeal carcinoma. *Cancer Letters*.

[2] Chua M. L. K., Wee J. T. S., Hui E. P., Chan A. T. C. (2016). Nasopharyngeal carcinoma. *The Lancet*.

[3] Ke L., Xiang Y., Xia W. (2016). A prognostic model predicts the risk of distant metastasis and death for patients with nasopharyngeal carcinoma based on pre-treatment interleukin 6 and clinical stage. *Clinical Immunology*.

[4] Zhao P., Fang W.-J., Chai L. (2015). The prognostic value of plasma soluble CD40 ligand levels in patients with nasopharyngeal carcinoma. *Clinica Chimica Acta*.

[5] Chan A. T., Hui E. P., Ngan R. K. (2018). Analysis of plasma epstein-barr virus dna in nasopharyngeal cancer after chemoradiation to identify high-risk patients for adjuvant chemotherapy: a randomized controlled trial. *Journal of Clinical Oncology*.

[6] Tsao S. W., Tsang C. M., Lo K. W. (2017). Epstein-Barr virus infection and nasopharyngeal carcinoma. *Philosophical Transactions of the Royal Society B: Biological Sciences*.

[7] Sharpe A. H., Pauken K. E. (2018). The diverse functions of the PD1 inhibitory pathway. *Nature Reviews Immunology*.

[8] Sorensen S. F., Demuth C., Weber B., Sorensen B. S., Meldgaard P. (2016). Increase in soluble PD-1 is associated with prolonged survival in patients with advanced EGFR-mutated non-small cell lung cancer treated with erlotinib. *Lung Cancer*.

[9] Wei K.-R., Zheng R.-S., Zhang S.-W., Liang Z.-H., Li Z.-M., Chen W.-Q. (2014). Nasopharyngeal carcinoma incidence and mortality in China in 2010. *Chinese Journal of Cancer*.

[10] Baumforth K. R., Birgersdotter A., Reynolds G. M. (2008). Expression of the epstein-barr virus-encoded epstein-barr virus nuclear antigen 1 in Hodgkin's lymphoma cells mediates up-regulation of Ccl20 and the migration of regulatory T cells. *The American Journal of Pathology*.

[11] Chung G. T., Lou W. P., Chow C. (2013). Constitutive activation of distinct NF-kappaB signals in EBV-associated nasopharyngeal carcinoma. *The Journal of Pathology*.

[12] Ressing M. E., van Gent M., Gram A. M., Hooykaas M. J., Piersma S. J., Wiertz E. J. (2015). Immune evasion by Epstein-Barr virus. *Current Topics in Microbiology and Immunology*.

[13] Kanakry J. A., Hegde A. M., Durand C. M. (2016). The clinical significance of ebv DNA in the plasma and peripheral blood mononuclear cells of patients with or without ebv diseases. *Blood*.

[14] Prayongrat A., Chakkabat C., Kannarunimit D., Hansasuta P., Lertbutsayanukul C. (2017). Prevalence and significance of plasma Epstein-Barr Virus DNA level in nasopharyngeal carcinoma. *Journal of Radiation Research*.

[15] Nielsen C., Ohm-Laursen L., Barington T., Husby S., Lillevang S. T. (2005). Alternative splice variants of the human PD-1 gene. *Cellular Immunology*.

[16] Oweida A., Lennon S., Calame D. (2017). Ionizing radiation sensitizes tumors to PD-L1 immune checkpoint blockade in orthotopic murine head and neck squamous cell carcinoma. *OncoImmunology*.

[17] Zhuang Y., Li S., Wang H., Pi J., Xing Y., Li G. (2018). PD-1 blockade enhances radio-immunotherapy efficacy in murine tumor models. *Journal of Cancer Research and Clinical Oncology*.

[18] Maity A., Mick R., Huang A. C. (2018). A phase I trial of pembrolizumab with hypofractionated radiotherapy in patients with metastatic solid tumours. *British Journal of Cancer*.

[19] Nagasaka M., Zaki M., Kim H. (2016). PD1/PD-L1 inhibition as a potential radiosensitizer in head and neck squamous cell carcinoma: a case report. *Journal for ImmunoTherapy of Cancer*.

[20] Pfannenstiel L. W., McNeilly C., Xiang C. (2018). Combination PD-1 blockade and irradiation of brain metastasis induces an effective abscopal effect in melanoma. *OncoImmunology*.

[21] Chen Z., Hu K., Feng L. (2018). Senescent cells re-engineered to express soluble programmed death receptor-1 for inhibiting programmed death receptor-1/programmed death ligand-1 as a vaccination approach against breast cancer. *Cancer Science*.

[22] Shin S.-P., Seo H.-H., Shin J.-H. (2013). Adenovirus expressing both thymidine kinase and soluble PD1 enhances antitumor immunity by strengthening CD8 T-cell response. *Molecular Therapy*.

[23] Chang B., Huang T., Wei H. (2019). The correlation and prognostic value of serum levels of soluble programmed death protein 1 (sPD-1) and soluble programmed death-ligand 1 (sPD-L1) in patients with hepatocellular carcinoma. *Cancer Immunology, Immunotherapy*.

[24] Kruger S., Legenstein M., Rösgen V. (2017). Serum levels of soluble programmed death protein 1 (sPD-1) and soluble programmed death ligand 1 (sPD-L1) in advanced pancreatic cancer. *OncoImmunology*.

[25] Long L., Zhang X., Chen F. (2018). The promising immune checkpoint LAG-3: from tumor microenvironment to cancer immunotherapy. *Genes Cancer*.

[26] Imai D., Yoshizumi T., Okano S. (2019). IFN-gamma promotes epithelial-mesenchymal transition and the expression of PD-L1 in pancreatic cancer. *Journal of Surgical Research*.

[27] Lamano J., Lamano J., Choy W. (2017). Glioblastoma-derived IL-6 induces immunosuppressive peripheral myeloid cell PD-L1 and promotes tumor growth. *Clinical Cancer Research*.

[28] Autio K., Oft M. (2019). Pegylated interleukin-10: clinical development of an immunoregulatory cytokine for use in cancer therapeutics. *Current Oncology Reports*.

